# Integrating network pharmacology with pharmacological research to elucidate the mechanism of modified Gegen Qinlian Decoction in treating porcine epidemic diarrhea

**DOI:** 10.1038/s41598-024-70059-5

**Published:** 2024-08-15

**Authors:** Jinzhong Cui, Xuehua Li, Yu Kang, Peng Li, Xinling Guo, Wei Zhao, Li Yang, Qinxin Yang, Ru Li, Xingyou Liu, Zilong Sun

**Affiliations:** 1 College of Veterinary Medicine, Shanxi Agricultural University, Mingxian South Road 1, Jinzhong, 030801 Shanxi China; 2 School of Medicine, Xinxiang University, Xinxiang, 453003 China; 3 School of Biological Engineering, Xinxiang University, Jinsui Road 191, Xinxiang, 453003 China; 4 School of Pharmacy, Xinxiang University, Xinxiang, 453003 China

**Keywords:** Porcine epidemic diarrhea virus, Modified Gegen Qinlian decoction, Network Pharmacology, IPEC-J2 cells, IFN-β, Pharmacology, Immune evasion, Inflammation

## Abstract

Porcine Epidemic Diarrhea Virus (PEDV) poses a significant threat to neonatal piglets, particularly due to the limited efficacy of existing vaccines and the scarcity of efficacious therapeutic drugs. Gegen Qinlian Decoction (GQD) has been employed for over two millennia in treating infectious diarrhea. Nonetheless, further scrutiny is required to improve the drug’s efficacy and elucidate its underlying mechanisms of action. In this study, a modified GQD (MGQD) was developed and demonstrated its capacity to inhibit the replication of PEDV. Animal trials indicated that MGQD effectively alleviated pathological damage in immune tissues and modulated T-lymphocyte subsets. The integration of network analysis with UHPLC-MS/MS facilitated the identification of active ingredients within MGQD and elucidated the molecular mechanisms underlying its therapeutic effects against PEDV infections. In vitro studies revealed that MGQD significantly impeded PEDV proliferation in IPEC-J2 cells, promoting cellular growth via virucidal activity, inhibition of viral attachment, and disruption of viral biosynthesis. Furthermore, MGQD treatment led to increased expression levels of IFN-α, IFN-β, and IFN-λ3, while concurrently decreasing the expression of TNF-α, thereby enhancing resistance to PEDV infection in IPEC-J2 cells. In conclusion, our findings suggest that MGQD holds promise as a novel antiviral agent for the treatment of PEDV infections.

## Introduction

Serious watery diarrhea, vomiting, dehydration, heightened mobidity, and mortality are typical clinical symptoms of porcine epidemic diarrhea (PED) caused by PEDV, an enteropathogenic intestinal alphacoronavirus. PEDV infection has resulted in heavy financial losses to the pig husbandry industry^1^. In recent years, epidemiological studies and analyses of PEDV infection have shown high positive detection rates for single, double, and triple infections, indicating that PEDV remains one of the major pathogens responsible for outbreaks in pig farms in China^2,3^. PEDV affects porcine intestinal epithelial cells (IECs) and inhibits the expression of type I and type III interferon (IFN) through various mechanisms, thereby evading the innate immune response^4–6^. Interferons (IFNs) are cytokines with antiviral, antitumor, and immunomodulatory effects. During viral infection, both IFN-I and IFN-III trigger the copying of IFN-stimulated genes (ISGs) through the JAK/STAT pathway, leading to strong and effective antiviral responses^7,8^. IFN-I exerts its antiviral functions in intestinal lymphocytes, while IFN-λ functions primarily in IECs^9^. These findings underscore the significance of IFN-I and IFN-λ in the host’s nonspecific immune response and their implications for screening drugs for PED treatment.

Traditional Chinese medicine (TCM) has gained widespread clinical recognition as an effective approach to treating various diseases. Previous studies have demonstrated that TCM can serve as a prophylactic or antiviral agent by enhancing the host’s innate immunity^10^. Gegen Qinlian decoction (GQD), a classical TCM formula, is used for treating various forms of infectious diarrhea related to damp-heat syndrome^11^. GQD has been shown to possess diverse pharmacological properties, including antipyretic, antibacterial, and antiviral effects, inhibition of gastrointestinal movements, and enhancement of immune function^12,13^. Novel veterinary pharmaceuticals derived from GQD such as Jinge Zhili powder, Gegen Lianqin powder, and Shuangge Zhixie oral liquid (Quality standards for veterinary drugs: Traditional Chinese Medicine Volume, 2017) have been employed to prevent and treat PED. However, the efficacy of these drugs has not met production requirements, underscoring the urgency of developing new environmentally friendly, economical, and efficient drugs to mitigate the losses inflicted by PED on the pig industry.

Network pharmacology emphasizes the interactions among drugs, genes, targets, and diseases from a holistic perspective that comprises biological networks and system-level balance^14^. The approach provides valuable technological and scientific support for clinical use and new drug discovery^14^. At the core of network pharmacology are “network targets” that encompass key molecules, pathways, and modules^15,16^. In this study, we utilized network pharmacology to analyze the bioactive compounds, core targets, and key pathways involved in the synergistic effects and mechanisms of modified Gegen Qinlian decoction (MGQD). Key molecules such as TNF-α, IFN-α, IFN-β, and IFN-λ that play pivotal regulatory roles in the pathogenesis of PED were identified. We then conducted a comprehensive pharmacological study to assess the anti-PEDV activity of MGQD from multiple perspectives.

## Results

### Effects of MGQD on immunosuppression

#### MGQD relieved immunosuppression

**Figure 1:**
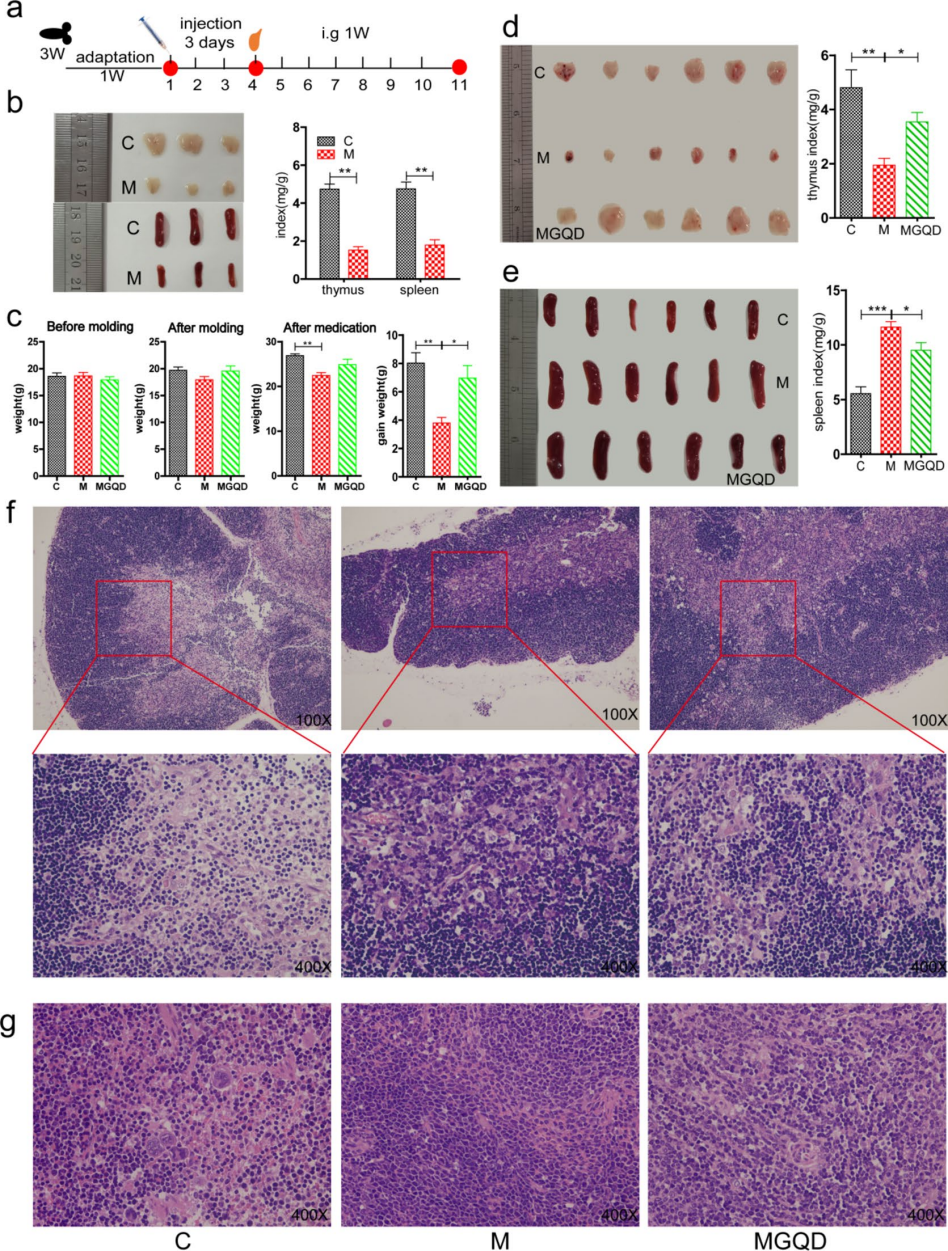
The effects of MGQD on immunosuppression. (**a**) Flow chart of the mouse immunodeficiency model. The red circular dots represent the weighing time nodes (i.e., the 1st, 4th, and 11th days), fasting for 12 h before weighing and drinking freely. (**b**) Three mice were randomly selected from the C and M groups for dissection after the completion of the model treatment. The thymus and spleen of group M mice showed significant atrophy. thymus index (mg/g) = thymus weight (mg)/ weight (g); spleen index (mg/g) = thymus weight (mg) /weight (g). (**c**) Comparison of body weight before and after modeling and after medication in each group. Gain weight (g) = body weight after medication (g)—body weight after modeling. (**d**, **e**) All experimental mice were dissected after the medication was completed. *P < 0.05, **P < 0.01, ***P < 0.001. (**f**, **g**) Histopathological changes in thymus and spleen in immunosuppressed mice. C: blank control group, M: cyclophosphamide (CTX)-induced model group, MGQD: MGQD treatment group.

A mouse immunodeficiency model was applied to validate the regulatory effect of MGQD on immunosuppression, since PEDV can evade the innate immune system. After three days of continuous intraperitoneal injection of CTX, three mice were randomly chosen from both group C and group M for dissection. Comparative analysis revealed that the thymus and spleen of Group M mice showed significant atrophy. The thymus and spleen indices were notably lower than those of group C, confirming the successful establishment of the immunodeficiency model (Fig. 1b).

Further analysis of body weight and immune organ indices revealed that compared with Group M, the MGQD group displayed a significant increase in weight gain (Fig. 1c) (P < 0.05). In addition, the MGQD group exhibited an increase in thymus size and a significantly higher thymus index (Fig. 1d) (P < 0.05). Notably, the spleen returned to its normal size and shape, and there was a significant reduction in the spleen index (Fig. 1e; P < 0.05). The results of HE staining (Fig. 1f, g) identified apparent pathological features, with medulla atrophy of the thymus and uneven cortical texture, spleen inflammation, increased reticulocytes, and decreased lymphocytes in group M. However, in the MGQD group, the histological features of the thymus and spleen structure were ameliorated, becoming typical of normal structures or demonstrating mild structural damage. Therefore, the autopsy findings and histopathological changes indicated that MGQD could relieve the immunosuppressed status.

#### MGQD regulated subsets of T-lymphocyte

**Figure 2:**
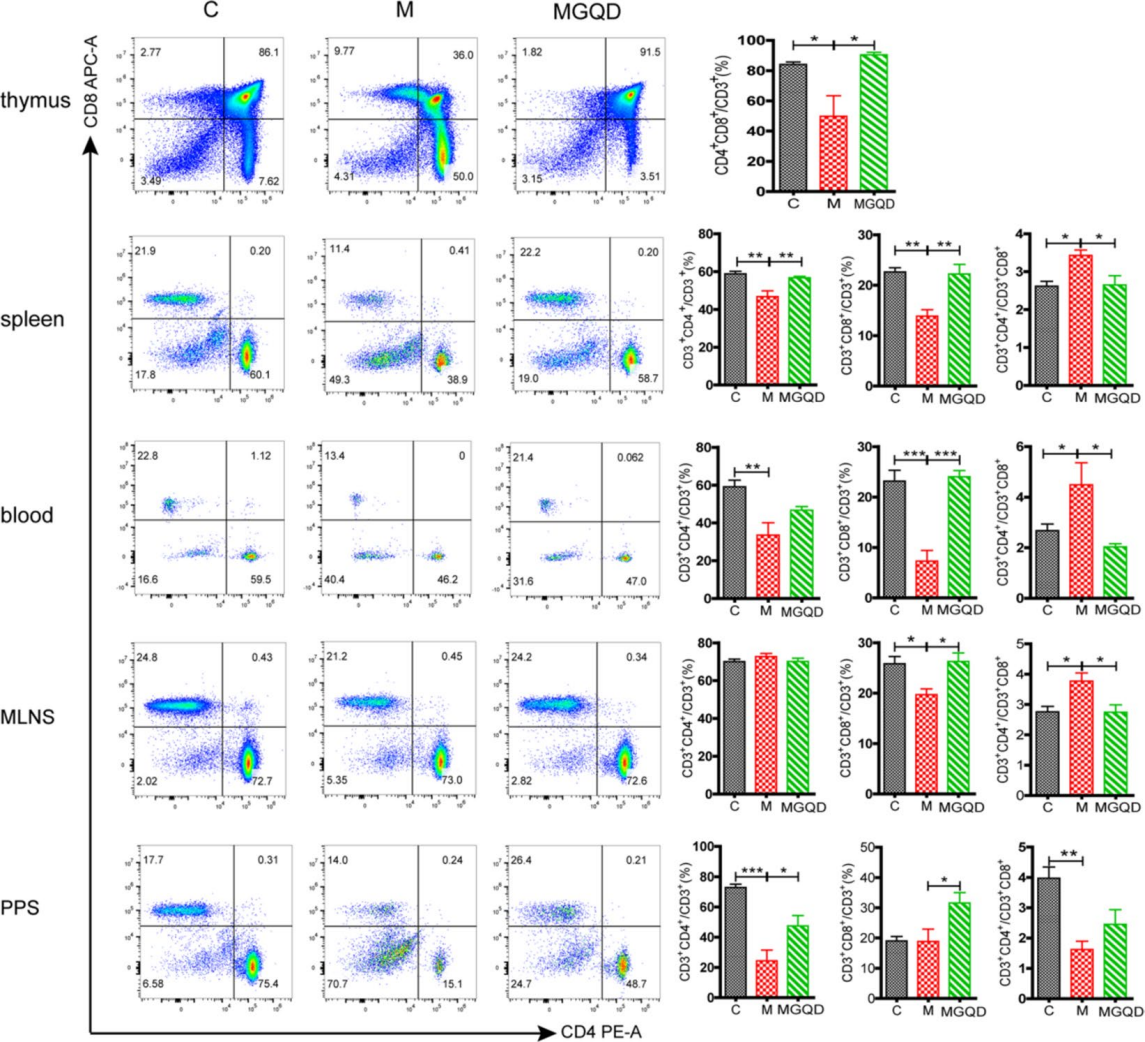
The effects of MGQD on subsets of T-lymphocyte in immunosuppressed mice. Obtain data on the CytoFLEX S system and analyze using FlowJoTM Software v10.8.1. C: blank control group, M: cyclophosphamide (CTX)-induced model group, MGQD: MGQD treatment group.*P < 0.05, **P < 0.01, ***P < 0.001.

To further evaluate MGQD’s regulatory impact on immune function, T-lymphocyte subsets were assessed using FCM. As shown in Fig. 2, compared with group C, the M group displayed notably reduced production of CD^3+^CD^4+^CD^8+^ in the thymus, CD^3+^CD^4+^ in the spleen, blood, and PPs, as well as CD^3+^CD^8+^ in the spleen, blood, and MLNs. Consequently, the CD^3+^CD^4+^/CD^3+^CD^8+^ ratio was elevated in the spleen, blood, and MLNs but reduced in the PPs. Conversely, compared with the M Group, MGQD administration resulted in a substantially higher ratio of CD^3+^CD^4+^CD^8+^ in the thymus, CD^3+^CD^4+^ in the spleen and PPs, and CD^3+^CD^8+^ in the spleen, blood, MLNs, and PPs. Furthermore, the CD^3+^CD^4+^/CD^3+^CD^8+^ ratio in the spleen, blood, and MLNs was significantly lower. These FCM results collectively suggested that MGQD effectively regulated the host’s immunosuppressive state and enhanced overall immunity.

### Potential mechanisms of MGQD against PED

#### Component identification of MGQD

**Figure 3:**
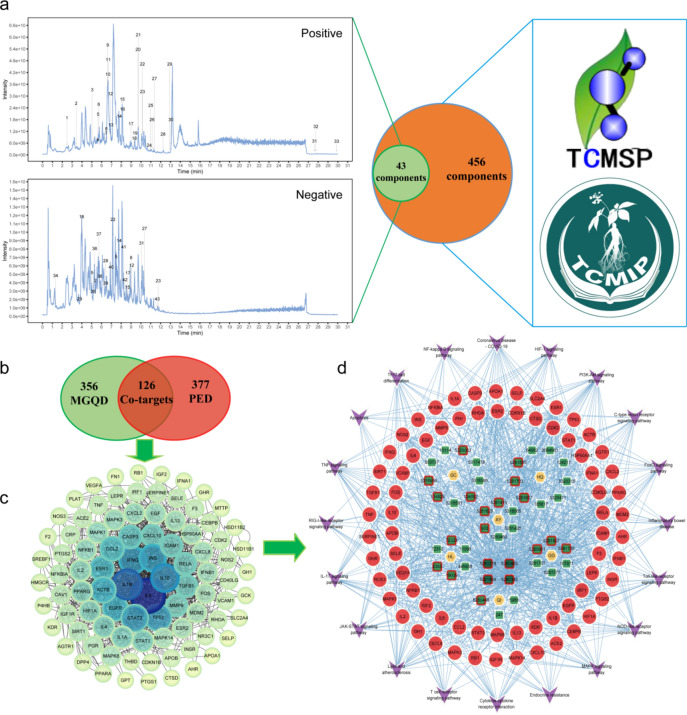
Network analysis of MGQD in the treatment of PED. (**a**) 43 main bioactive components in MGQD were identified using UHPLC-MS/MS; 456 components within MGQD were screened from databases; 43 components were ultimately selected as representative components of MGQD. (**b**) Venn diagram of 126 co-targets of MGQD and PED. (**c**) The 90 main targets were extracted from 126 co-targets via PPI network analysis. (**d**) Interaction network of drugs − components − targets − pathways. GG: Puerariae Lobatae Radix, HQ: Scutellariae Radix, HL: Coptidis Rhizoma, QI: Astragali Radix, GC: Glycyrrhizae Radix Et Rhizoma Praeparata Cum Melle, AY: Artemisiae Argyi Folium. Yellow nodes represent herbs in MGQD; green nodes represent components; red nodes represent targets, and purple nodes represent signaling pathways. The quadrilateral with a red border represents 21 identified core components based on the degree value. Among them, CID5280343 is a common component of HL, GC, QI and AY; CID5280378 is a common component of GG, GC and QI; CID5280863 and CID5281654 are common components of GC and QI.

UHPLC-MS/MS was used to identify the components of MGQD and to establish a quality control for MGQD. Typical ion flow and base peak chromatographs are shown in Fig. 3a. According to the standards and relational databases, 43 core components from MGQD were initially identified (Fig. 3a). The classes of components included flavonoids, phenylpropanoids, alkaloids, terpenoids, chalcones, and organooxygen compounds. Detailed characterizations of each component are showed in Table S1.

#### Obtaining data for MGQD and PED-associated targets

Following the established screening criteria, a set of 456 chemical components of MGQD were successfully acquired from the databases. Then, 43 main components were gained through the intersection of results from UHPLC-MS/MS and the above databases (Fig. 3a). A total of 482 prediction targets associated with these 43 core components were obtained. In addition, 503 disease-related targets closely associated with PED were obtained. After further comparison and screening, a refined selection of 126 common targets shared by both MGQD and PED was obtained (Fig. 3b). Detailed information concerning the MGQD components and presumed targets is provided in Tables S2 and S3.

#### PPI network construction

The PPI network information for the interaction between MGQD and PED was got by importing the 126 common targets into the STRING12.0 database. This resulted in a network comprising 124 nodes and 405 edges. The average degree of node was 6.53, with an expected number of edges of 66 and a PPI enrichment *P* value of less than 1.0e–16. During this visualization, a total of 90 targets were extracted based on a degree value ≥ 2. Among these, 32 targets had a degree value ≥ 20, including well-known targets such as IL6, EGFR, STAT1, and IFNB1 (Fig. 3c, Table S4). This observation underscores the intricate and multifaceted nature of the mechanisms underlying the treatment of PED with MGQD.

#### Functional enrichment analysis

**Figure 4:**
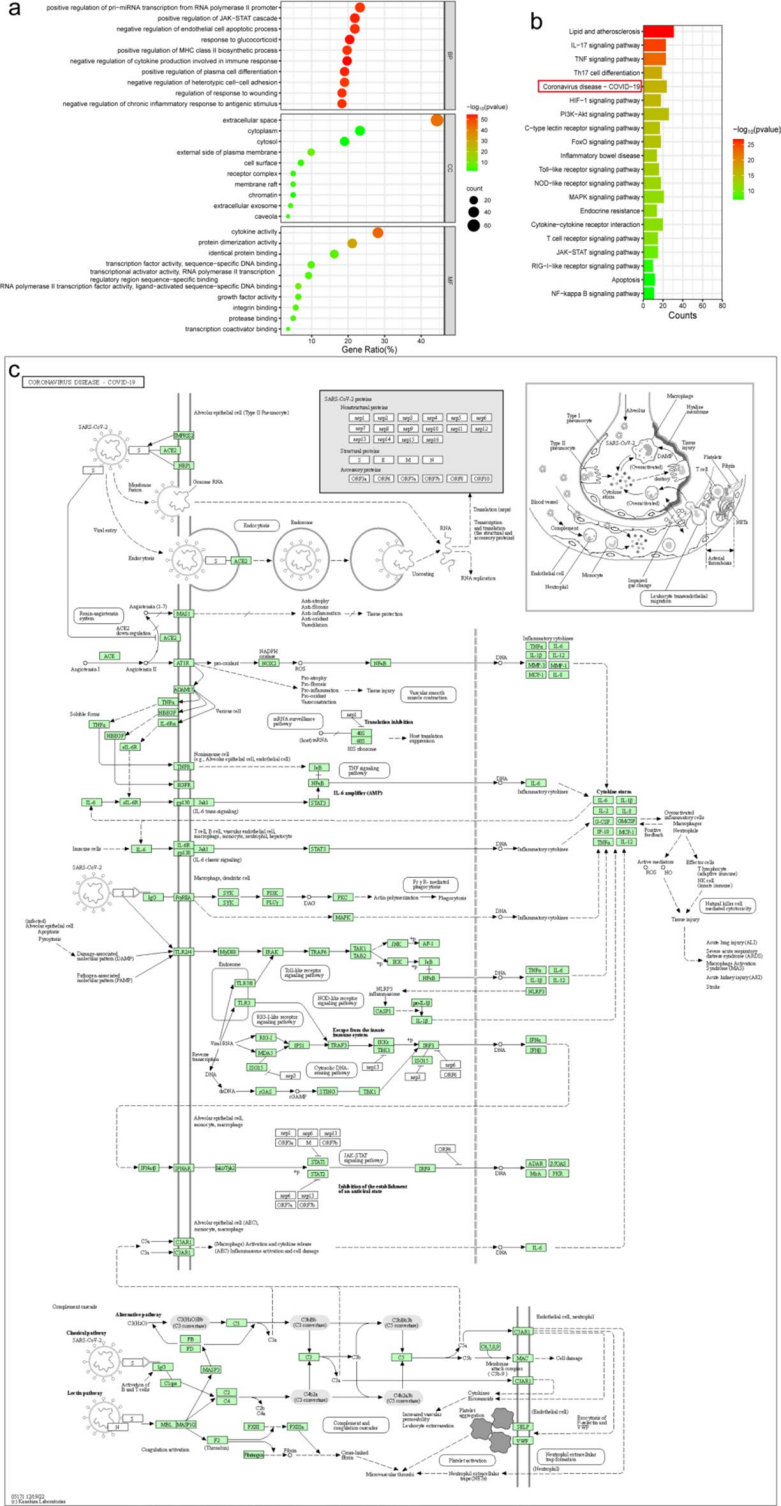
Enrichment analysis of 90 main targets. (**a**) GO functional analysis bubble diagram, and respectively displayed the top 10 enriched terms in BP, CC and MF. (**b**) KEGG enrichment pathway bar chart, and displayed the top 20 of disease-related pathways by the Pvalue of significance. The pathway with red border is the pathway studied in this article. (**c**) Coronavirus disease signaling pathway^40–42^.

A GO function analysis conducted on 90 targets revealed significant enrichment with 331 chart records (*P* < 0.05), encompassing 263 BPs, 20 CCs, and 48 MFs as determined by the David database. The top 10 enriched BP terms encompassed a range of functions, including negative regulation of cytokine production involved in immune response, response to glucocorticoids, positive regulation of plasma cell differentiation, and positive regulation of JAK-STAT cascade. The top 10 terms in CC included extracellular space, external side of plasma membrane, and extracellular exosome. The top 10 MF terms were primarily associated with cytokine activity, protein dimerization activity, and growth factor activity (Fig. 4a, Table S5).

Furthermore, 148 KEGG enrichment pathways were screened according to the criterion of FDR < 0.05. Among these pathways, the top 20 in terms of disease-related pathways were highlighted by their significant FDR values (Fig. 4b). These included the Coronavirus disease-COVID-19, the TNF signaling pathway, the JAK-STAT signaling pathway, the NF-kappa B signaling pathway, and the RIG-I-like receptor signaling pathway. Therefore, multiple active compounds within MGQD may exert therapeutic effects on PED through these signaling pathways. Notably, the crucial mechanisms responsible for the effects of MGQD in the treatment of PED may be related to the Coronavirus disease signaling pathway (Fig. 4c). This is primarily because the Coronavirus disease signaling pathway not only encompasses numerous disease-related signaling pathways, such as TNF signaling pathway, Toll-like receptor signaling pathway, NOD-like receptor signaling pathway, RIG-I-like receptor signaling pathway, JAK-STAT signaling pathway, NF-kappa B signaling pathway, etc., but also because the pathogenic mechanism of coronavirus mainly involves viral evasion of the innate immune system and inhibition of the establishment of an antiviral state. We have demonstrated that MGQD could effectively alleviated the immunosuppressive state and enhanced immunity through CTX-induced mouse immunosuppression assay. Therefore, we speculate that the key mechanism of MGQD in treating PED may be related to the Coronavirus disease signaling pathway.

An interaction network involving “drug − active compounds − targets − pathways − 

disease” was established (Fig. 3d). The intricate nature of this network highlights the potential for various components and targets within MGQD to interact and collectively influence the broader biological network, a characteristic commonly observed in traditional Chinese medicines.

### Molecular docking analysis

**Figure 5:**
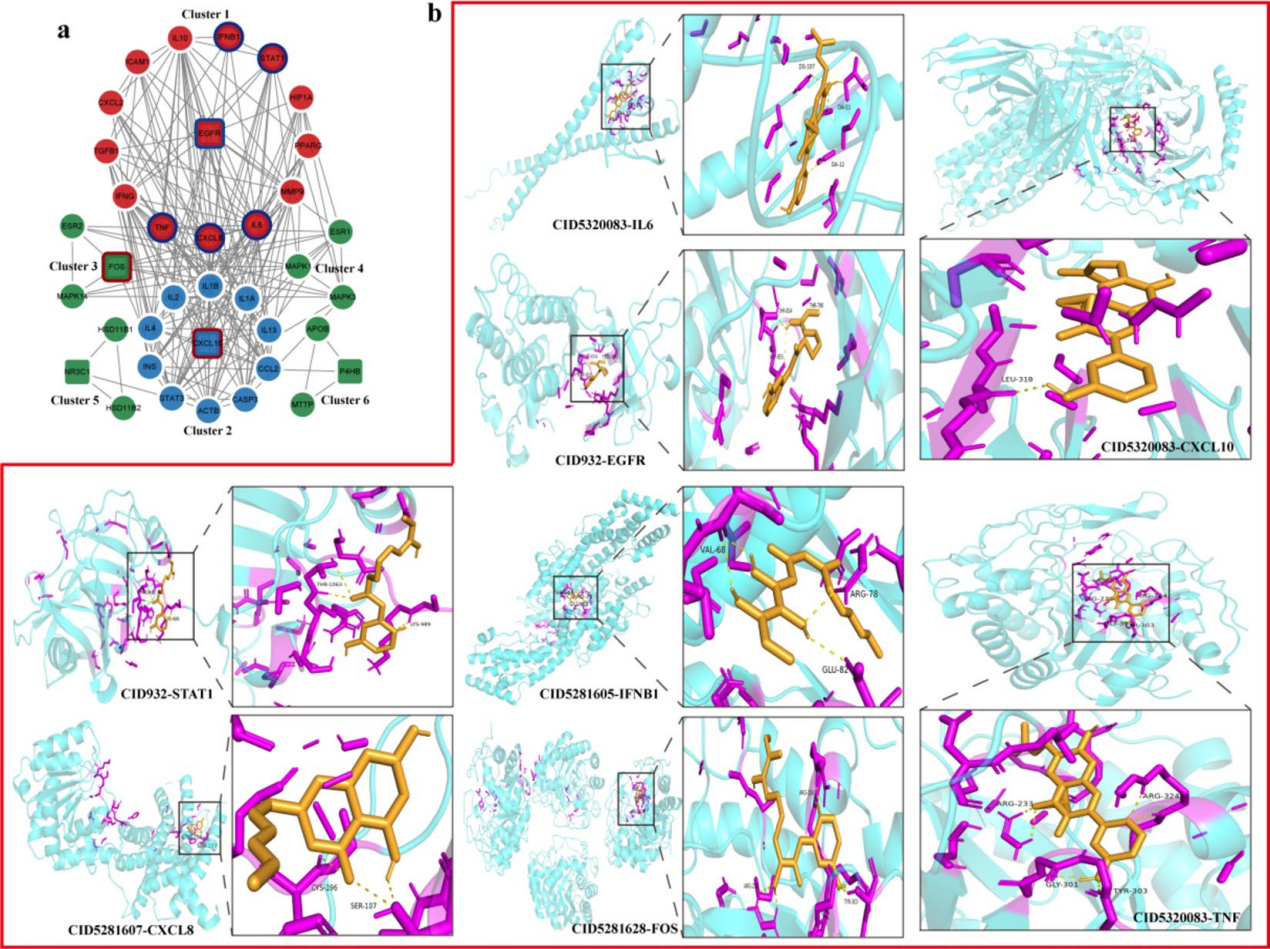
Modular analysis of the main targets and molecular docking. (**a**) Obtained six significant clusters via the MCODE plugin. Square nodes represent seed genes; circular nodes represent clustered genes; genes with border represent key targets. (**b**) Representative molecular docking complex of each key target and components.

To further probe the underlying mechanism, six significant clusters and eight key targets were generated via the MCODE plugin (Fig. 5a, Table S6). Validation of the interaction between 21 identified core components (Fig. 3d) and eight kernel targets was performed through molecular docking. As shown in Table S7 and Fig. 5b, the strongest binding ability shown by pairs of identified core compounds and kernel targets was as follows: IL6 to CID5320083 (–8.7 kcal/mol), EGFR to CID932 (–10.6 kcal/mol), CXCL10 to CID5320083 (–9.4 kcal/mol), STAT1 to CID932 (–7.7 kcal/mol), IFNB1 to CID5281605 (–9 kcal/mol), CXCL8 to CID5281607 (–7.9 kcal/mol), FOS to CID5281628 (–9 kcal/mol), and TNF to CID5320083 (–10.6 kcal/mol). This verification of molecular docking implied that core chemical components of MGQD had a strong binding activity to the key targets.

### Cytotoxicity of MGQD to IPEC-J2 cells

**Figure 6:**
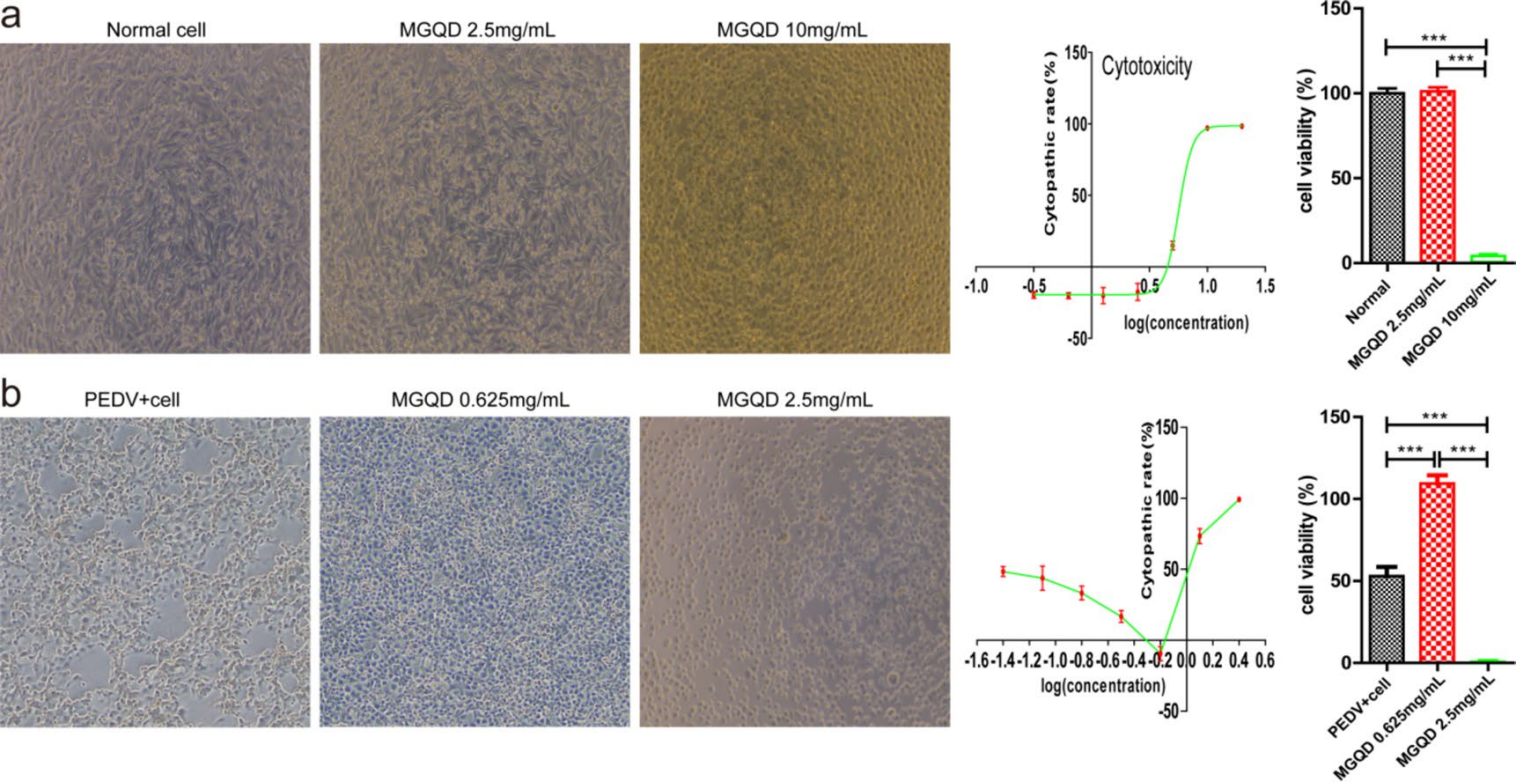
Determination of cytotoxicity and action concentrations of MGQD separately. (**a**) Cytotoxicity and TC50 curve of MGQD on IPEC-J2 cells. The cytotoxicity curve showed that within a certain concentration range, cytotoxicity increases with the increase of drug concentration. Morphology of IPEC-J2 cells (100X). The bar chart indicated that the activity of IPEC-J2 cells was significantly decreased when the concentration of MGQD was 10 mg/ml; whereas at a concentration of 2.5 mg/ml, there was no significant difference in cell activity compared to the normal cell group. (**b**) Determination and curve plot of suitable concentration of MGQD for IPEC-J2 cells infected with PEDV. The curve showed that the drug was only suitable for acting on IPEC-J2 cells infected with PEDV within a certain concentration range. Morphology of IPEC-J2 cells (40X). The bar chart demonstrated that the activity of PEDV-infected cells was significantly reduced, while 0.625 mg/ml MGQD significantly increased cells viability. *** P < 0.001.

Microscopic observations of the cytopathological effects (CPEs) were conducted on IPEC-J2 cells treated with various concentrations of MGQD (Fig. 6a). Notably, when cells were exposed to 10 mg/mL MGQD, they exhibited shedding and displayed circular or deceased morphology. By contrast, treatment with 2.5 mg/mL MGQD resulted in minimal changes to cell morphology. Subsequent optical density measurements were made using a microplate reader, and the rate of cytopathic damage was calculated.

GraphPad Prism™ 5.0 was utilized to generate a graph for TC50 and estimate its value (5.59 ± 0.16 mg/mL). Interestingly, drug concentrations that exhibited no toxicity toward normal IPEC-J2 cells demonstrated pronounced toxicity toward IPEC-J2 cells infected with PEDV. This observation indicated a significant reduction in the activity of IPEC-J2 cells infected with PEDV (Fig. 6b). Considering in light of this finding, a concentration of 0.625 mg/mL MGQD was selected as the appropriate dosage for subsequent experiments involving PEDV-infected cells (MOI = 0.1).

### Anti-PEDV effect of MGQD

**Figure 7:**
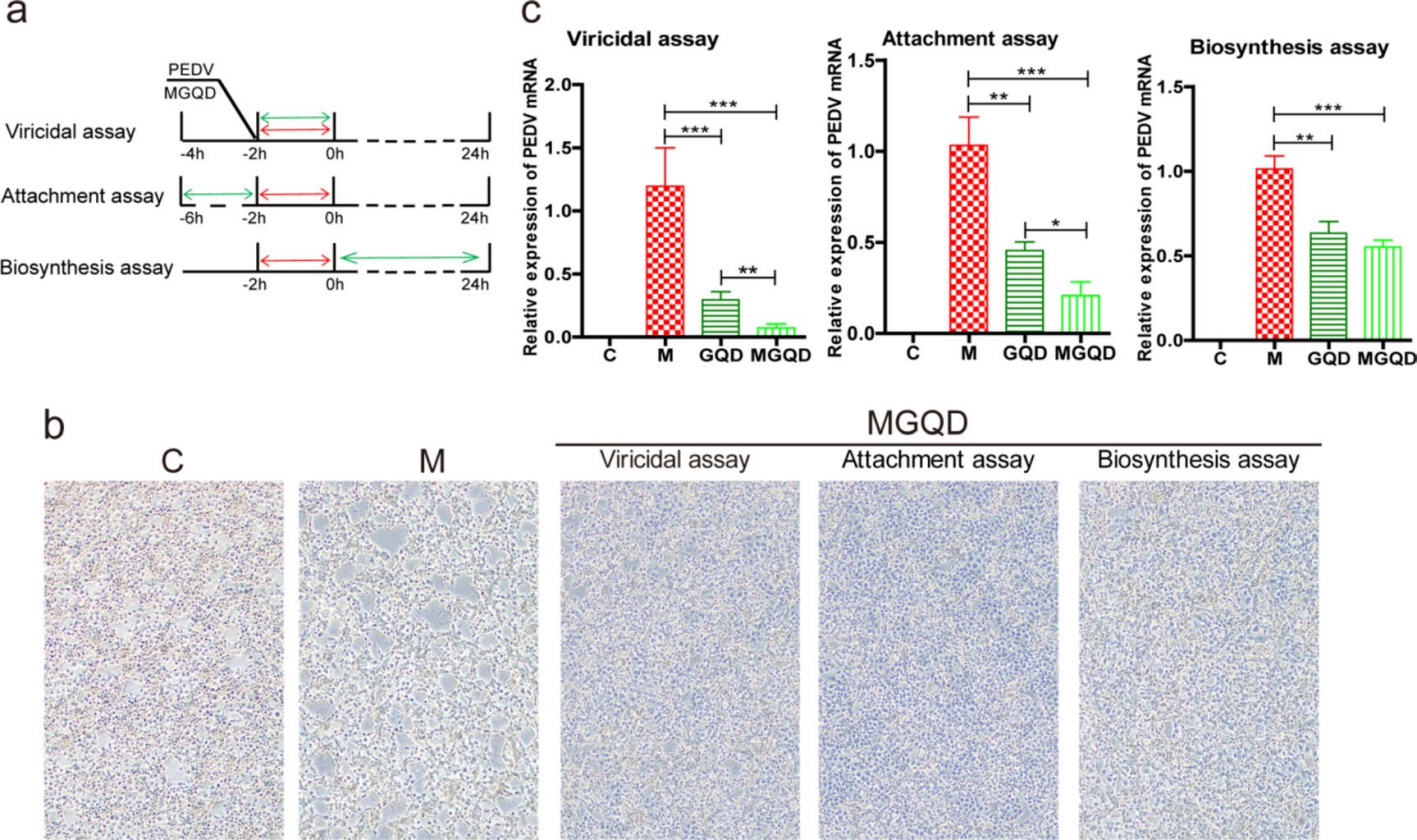
Anti-PEDV effect of MGQD. (**a**) Antiviral assays using three distinct aspects of infection: viricidal, attachment and biosynthesis. Green arrow represent MGQD, and red arrow represent PEDV. (**b**) Morphology of IPEC-J2 cells (40 ×). (**c**) The expression levels of PEDV mRNA. C: normal cells control group, M: PEDV-infected model group, GQD: GQD treatment group. MGQD: MGQD treatment group. *P < 0.05, **P < 0.01, ***P < 0.001.

To investigate the anti-PEDV effect of MGQD, we conducted antiviral assays utilizing three distinct aspects of infection: viricidal, attachment, and biosynthesis **(**Fig. 7a**).** We began by observing the morphology of IPEC-J2 cells under the microscope (Fig. 7b). In group C, the cells exhibited an intact and evenly distributed morphology. In group M, however, there were increases in cell fusion, abscission, and vacuoles. The MGQD-treated group displayed significantly enhanced cell growth, with a dense cell layer structure. The expression levels of PEDV mRNA were then assessed via RT‒qPCR (Fig. 7c). In the C group, the PEDV mRNA was not detected. By contrast, in group MGQD, the PEDV mRNA was notably reduced in all three antiviral tests compared with group M (*P* < 0.001). Meanwhile, MGQD had significantly higher efficacy in reducing viral load compared to GQD (*P* < 0.01, *P* < 0.05).The results collectively demonstrated that MGQD effectively impeded the proliferation of PEDV across various stages of the viral life cycle.

### Effects of MGQD on the protein and mRNA levels of IFN-α, IFN-β, IFN-λ3, and TNF-α in IPEC-J2 cells.

**Figure 8:**
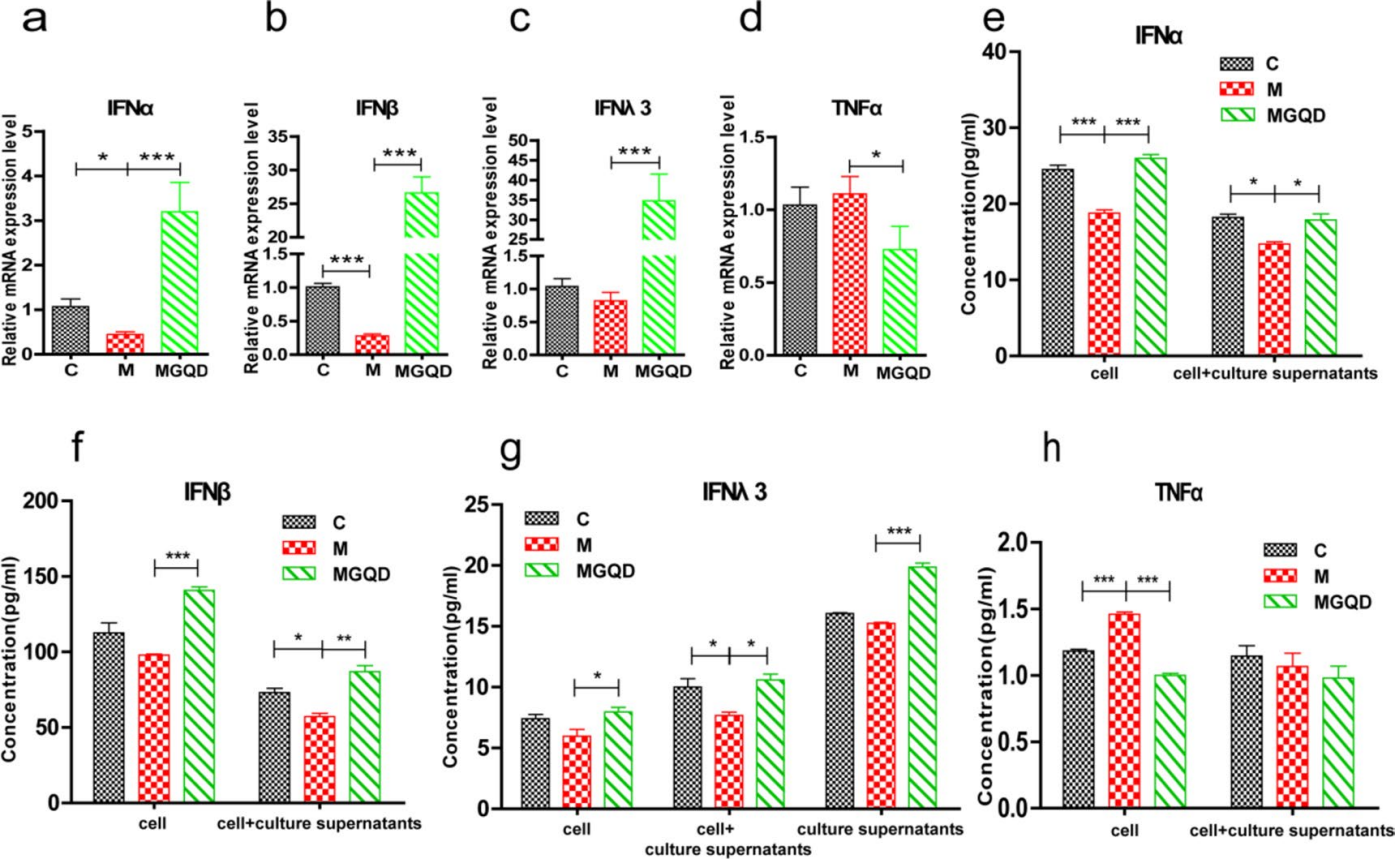
The effects of MGQD on the mRNA and protein levels of IFN-α, IFN-β, IFN-λ3, and TNF-α in IPEC-J2 cells. (**a**–**d**) The expression levels of IFN-α, IFN-β, IFN-λ3 and TNF-α mRNA. (**e**–**h**) The expression levels of IFN-α, IFN-β, IFN-λ3 and TNF-α protein were detected by ELISA. The statistical results of TNF-α are the outcomes obtained after concentrating the sample and conducting an ELISA test for conversion. C: normal cells control group, M: PEDV-infected model group, MGQD: MGQD treatment group. *P < 0.05, **P < 0.01, ***P < 0.001.

The mRNA levels of IFN-α and IFN-β in the M group were markedly reduced compared with those in the C group (*P* < 0.05, *P* < 0.001) **(**Fig. 8a–d**).** Conversely, in the MGQD-treated group, the mRNA levels of IFN-α, IFN-β, and IFN-λ3 were significantly elevated compared with the model group (*P* < 0.001), while the mRNA of TNF-α was significantly lower (*P* < 0.05).

To attain a more precise understanding of MGQD’s anti-PEDV effects, ELISA was employed to measure the protein concentrations of IFN-α/β/λ3 and TNF-α in cell culture supernatants, cell lysates, and their mixtures. As shown in Fig. 8e–h, compared to the control group, the protein concentration of IFN-α in the cell lysate of group M was significantly diminished (*P* < 0.001), while the TNF-α level was significantly elevated (*P* < 0.001). Moreover, the IFN-α, IFN-β, and IFN-λ3 protein levels in the mixtures were all significantly reduced (*P* < 0.05). Conversely, in the MGQD group, the protein concentrations of IFN-α, IFN-β, and IFN-λ3 in both cell lysates and mixtures were substantially increased. Meanwhile, the TNF-α level in the cell lysate was significantly diminished (*P* < 0.001). Notably, the IFN-λ3 protein level in the cell culture supernatant was markedly increased (*P* < 0.001).

The combined findings from RT‒qPCR and ELISA consistently demonstrated that MGQD substantially enhanced the expression of IFN-α, IFN-β, and IFN-λ3 while concurrently reducing the level of TNF-α, an inflammatory factor. This dual effect contributed to the reinforcement of the innate immune response, ultimately leading to the manifestation of anti-PEDV effects.

## Discussion

The ongoing emergence of highly pathogenic variants of PEDV has posed a significant threat to the swine industry, underscoring the need for more effective antiviral solutions due to the limitations of current commercial PEDV vaccines^17,18^. Existing vaccines have not demonstrated ideal immune effects, highlighting the urgency of developing improved antiviral drugs to address the gaps left by vaccination strategies. TCM has garnered increasing recognition for its multitarget and multilevel functional effects, making it a promising avenue for research and development^15,19^. The classical formula of GQD has been used for treating infectious diarrhea for over two millennia^12^. Our prior investigations have demonstrated that MGQD, a variation of GQD, exhibits enhanced pharmacological effects, including antibacterial and antidiarrheal properties. In this context, we embarked on a comprehensive pharmacological study to explore the potential of MGQD for treating PEDV. Such research carries significant implications for the advancement of novel interventions to prevent and treat PEDV infections while bolstering immune function.

Given the intricate nature of MGQD’s components, our approach began by employing UHPLC-MS/MS to identify the active compounds. We identified 43 main active components from MGQD. In comparisons of the prediction targets of these 43 main components with PED-related targets, 90 central targets were extracted via PPI topological analysis. Subsequent functional analysis of these 90 central targets further demonstrated that MGQD’s active compounds primarily modulated cytokine production involved in immune responses, plasma cell differentiation, the JAK-STAT cascade, endothelial cell apoptotic processes, inflammatory responses, cytokine activity, and more. In addition, we constructed single drug-compound-target-pathway networks, and an analysis of the network revealed 21 identified core active compounds within MGQD that were involved in the effect against PED (Fig. 3d and Table S1). The above results provide a relatively comprehensive macroscopic mechanistic perspective for MGQD treatment of PED. To further explore the underlying mechanism in greater detail, we refined the classification and examined the interactions among the 90 targets via MCODE algorithm. The central targets were separated into six clusters, and eight key targets were selected (Fig. 5a). Validation of the interaction between 21 screened core compounds (Fig. 3d) and eight key targets was performed via molecular docking. The results showed that the identified core compounds of MGQD had a good or strong binding steadily to all the key targets. CID5281607, CID2353, CID5320083, CID5281703, CID5280448, CID932, and CID5281628 were considered significant components of MGQD for treating PED. Collectively, these findings suggest that MGQD may enhance interferon production, suppress inflammation, regulate cell apoptosis, and fortify the immune response through its influence on key targets, thereby serving as a candidate therapy for PEDV.

The results of the network pharmacology analysis were used to predict the network intervention and impact of MGQD on PED based on system biology analysis. We executed pharmacology experiments to validate the effect of MGQD in the treatment of PED. Recent studies have implicated subepithelial CD3^+^ T cells as transmission vectors for PEDV via air transmission in neonatal pigs, transferring the virus from an infected CD3^+^ T-cell to multiple uninfected counterparts^4,20^. Another study highlighted how PEDV exploits the chemokine CXCL-9 to attract specific T-cell subsets, thereby facilitating viral amplification and dissemination^21^. In our *in* vivo study, MGQD exhibited the ability to promote weight gain, increase immune organ growth, and ameliorate pathological damage of the thymus and spleen in immunosuppressed mice. Furthermore, MGQD significantly increased the production of CD3^+^CD4^+^CD8^-^, CD3^+^CD4^-^CD8^+^, and CD3^+^CD4^+^CD8^+^ T-lymphocyte subsets while also reducing the CD3^+^CD4^+^/CD3^+^CD8^+^ ratio. These results indicate that MGQD has the potential to alleviate immune suppression by comprehensively regulating the production and balance of T-cell subsets, thereby enhancing the innate immune defense system.

A major contributor to the heightened susceptibility of neonatal piglets to PEDV infection is the sluggish regeneration of IECs^22^. PEDV, exhibiting tissue and cell tropism, targets the small intestine of piglets, infecting IECs and replicating within the cytoplasm^6,23^. Pathological examination of PEDV-infected piglets revealed compromised intestinal villus structures, IEC necrosis, exfoliation from the lamina propria, nuclear concentration, and cytoplasmic vacuolization^24^. Our in vitro study observed similar CPEs in PEDV-infected IPEC-J2 cells, characterized by cell detachment and disruption of the cell layer structure^25^. However, MGQD treatment effectively reversed these CPEs, promoting denser cell layer structures in PEDV-infected IPEC-J2 cells. Notably, the PPI network analysis highlighted that epidermal growth factor (EGF) and its receptor EGFR were appropriate targets for MGQD’s PEDV treatment, both of which are associated with promoting the proliferation of intestinal crypt epithelial cells and mitigating atrophic enteritis induced by PEDV infection in nursing piglets^26^. This underscores MGQD’s potential to stimulate IPEC-J2 cell growth by modulating EGF and EGFR, thereby counteracting PEDV infection. Moreover, MGQD significantly reduced PEDV mRNA expression across the viricidal, attachment, and biosynthesis stages, underscoring its multifaceted inhibition of PEDV proliferation. In essence, these results collectively demonstrate that MGQD operates through multiple pathways to impede PEDV growth, demonstrating promising anti-PEDV effects.

In the context of neonatal pigs, another significant factor contributing to their heightened susceptibility to non-S INDEL PEDV infection is the immaturity of their gut innate immunity^27^. Following PEDV infection, the immediate damage to the intestinal villi disrupts the tight junctions between IECs, compromising the integrity of the intestinal epithelial barrier. This disruption in turn leads to impaired intestinal immune function and digestive dysfunction, exacerbating the vulnerability of neonatal piglets to PEDV infection^27,28^. Our KEGG enrichment analysis highlighted several core targets associated with pathways, including Coronavirus disease-COVID-19, the TNF signaling pathway, the JAK-STAT signaling pathway, RIG-I-like receptor signaling, Inflammatory bowel disease, NOD-like receptor signaling, and Toll-like receptor signaling. These pathways are involved in inflammation, the immune response, apoptosis, and immune deficiency. Previous research has established that PEDV employs multiple pathways to inhibit IFN-I and IFN-III responses, including pathways such as RIG-I-like receptor signaling and NF-kappa B signaling^29,30^, thus evading the host’s nonspecific immune response and counteracting the production of ISGs^5,31^. IFNs are cytokines known for their broad-spectrum antiviral and immune-regulatory effects. Both IFN-I and IFN-III activate the JAK-STAT signaling pathway, resulting in the production of ISGs that contribute to a robust antiviral response^7,32^. Notably, IFN-λ3 in IECs serves as a critical factor in natural antiviral immunity, collaborating with IFN-α/β to mount an effective antiviral response^9,33,34^. In our study, after PEDV infection of IPEC-J2 cells, both mRNA and protein generation of IFN-α, IFN-β, and IFN-λ3 were markedly reduced, while TNF-α protein levels exhibited a significant increase – a trend consistent with earlier reports^35,36^. Conversely, MGQD treatment substantially elevated the mRNA and protein generation of IFN-α, IFN-β, and IFN-λ3, concurrently reducing TNF-α protein levels. These observations suggest that MGQD may activate both type I and type III IFN signaling pathways to enhance IFN expression, thereby augmenting the body’s natural immune response against PEDV.

## Conclusions

In this study, a combination of network pharmacology and pharmacological research was employed to identify the principal bioactive constituents of MGQD and their interactions with numerous targets across multiple signaling pathways. As a result, MGQD demonstrated the ability to hinder the propagation of PEDV through a multifaceted mechanism. Notably, the observed enhancement of IEC growth and the modulation of innate immune responses contributed to the antiviral activity of MGQD. These findings offer valuable information that can inform the clinical application and further development of novel antiviral drugs aimed at treating PEDV infections.

## Materials and methods

### Preparation of MGQD

MGQD was prepared from extracts of six natural medicinal plants (Table S8). All Chinese medicinal materials were purchased from Zhang Zhongjing Pharmacy in Xinxiang City, Henan Province. The water extract of MGQD was prepared using the method described by the theory for typhoid miscellaneous diseases. Briefly, all herbs were soaked in 20 volumes of distilled water for 30 min. First, GG was boiled for 30 min, then mixed with the remaining herbs and again boiled for 30 min, and then these steps were repeated. Finally, the extract was filtered, combined, and concentrated to 57 ml to obtain a concentration of 1 g crude drug per ml. The concentrated decoction was sterilized and stored at 4 °C for future use.

### Components of MGQD

The chemical components of MGQD extract were identified using UHPLC-MS/MS. Briefly, the collected supernatant of the MGQD extract was analyzed on a UHPLC system (Vanquish, Thermo Fisher Scientific) with a Waters UPLC BEH C18 column (1.7 μm, 2.1*100 mm). The flow rate was set at 0.4 mL/min, and the sample injection volume was set at 5 μL. The mobile phase consisted of 0.1% formic acid (LC–MS grade solvents, CNW Technologies) in water (A) and 0.1% formic acid in acetonitrile (B) (LC–MS grade solvents, SIGMA). The multistep linear elution gradient program was as follows: 0 min, A:B = 95:5; 3.5 min, A:B = 85:15; 6 min, A:B = 70:30; 12 min, A:B = 30:70; 18 min, A:B = 0:100; 26–30 min, A:B = 95:5. In addition, an Orbitrap Exploris 120 mass spectrometer coupled with an Xcalibur software (Xcalibur, Thermo Fisher Scientific) was employed to acquire the MS and MS/MS data based on the IDA acquisition mode. During each acquisition cycle, the mass range was from 100 to 1500; the top four results of each cycle were sifted, and the corresponding MS/MS data were obtained. The sheath gas flow rate was 30 Arb; the Aux gas flow rate was 10 Arb; the Ion Transfer Tube Temp was 350°C; the Vaporizer Temp was 350°C; the Full ms resolution was 60,000; the MS/MS resolution was 15,000; the Collision energy was 16/32/48 in the NCE mode, and the Spray Voltage was 5.5 kV (positive) or –4 kV (negative).

### Immunoregulation assay

#### Animals

Animal experimentation was conducted following the approval and guidelines set by the Biological and Medical Ethics Committee of Xinxiang University. The dosage for mice was determined based on relevant references^37^. Three-week-old KM mice (License No.: SCXK (Beijing) 2021–0006) were obtained from Beijing Vital River Laboratory Animal Technology Co., Ltd. (Beijing, China) and were maintained under specific conditions (12-h light/dark cycle, 20–25°C, and relative humidity of 50 ± 10%). The mice had free access to food and water. After seven days of acclimatization, 24 mice were randomly assigned to three groups: a blank control group (C, n = 9), a model group (M, n = 9), and an MGQD group (MGQD, n = 6). Each group was individually housed in separate cages.

#### Flow cytometry (FCM)

The mice in the M and MGQD groups received intraperitoneal injections of 80 mg/kg cyclophosphamide (CTX) (Shanghai McLin Biochemical Technology Co., Ltd., Shanghai, China) for three consecutive days, establishing an immunodeficiency model^38^. Concurrently, the mice in the C group were injected with an equivalent volume of physiological saline. On the fourth day, three mice from each group (C and M) were selected at random and euthanized by cervical dislocation. Their tissues were dissected to verify the success of the model establishment. Starting from day 4, the MGQD group received an oral dose of MGQD (10 μL·g^–1^·d^–1^), while the C and M groups received an equal volume of water via oral gavage for seven days. The entire animal experiment (Fig. 1a) lasted for 11 days. The mice were weighed on days 1, 4, and 11 after a 12-h fasting period. On day 11, peripheral blood was collected from the eyeballs using EDTAK2 (Shandong Aosaite Medical Instruments Co., Ltd., Shanghai, China) as an anticoagulant. Subsequently, the mice were euthanized by cervical dislocation, and their half thymus, half spleen, mesenteric lymph nodes (MLNs), and Peyer’s patches (PPs) were dissected and processed into single-cell suspensions. The cells were stained with CD3, CD4, and CD8 monoclonal antibodies (BioLegend) to analyze T lymphocyte proliferation. Data were collected using the CytoFLEX S system (BECKMAN, USA) and analyzed using FlowJoTM Software v10.8.1 (Becton, Dickinson and Company, USA).

#### Thymus and spleen histology

The tissues of the thymus and spleen were fixed in 4% paraformaldehyde, embedded in paraffin, and sectioned at a thickness of 4 μm. The sections were stained with hematoxylin and eosin (HE). The histopathological changes were observed and photographed under a fluorescence microscope (DM1000, Leica, Germany).

### Network analysis of MGQD treatment for PED

The ingredients and ingredient-related targets within MGQD were identified via the databases of TCMSP (OB ≥ 30% and DL ≥ 0.18) and TCMIP (a confidence index of greater than 0.80 was used as the criterion). The identifications of targets related to PED were obtained from the NCBI and GeneCards databases. Then, the intersection of PED-associated targets with the MGQD ingredient targets was achieved using a Venn diagram. The shared targets were input into the STRING database for analysis, and the results were visualized using Cytoscape 3.9.1. GO and KEGG enrichment analyses of the shared targets were carried out via the David database, and the results were subsequently imported into the Online Bioinformatics platform for visualization.

### Molecular docking validation

The MCODE algorithm of Cytoscape was used for cluster analysis of the PPI network, and key targets of MGQD in the treatment of PED were screened through identifying highly connected regions. Then, the gene codes of the above-screened targets were selected from the UniProt database, and 3D structures of the key genes were obtained and modified as protein receptors utilizing the RCSB PDB database. Subsequently, 2D structures of the core MGQD chemical components were obtained as ligands from the PubChem database, and OpenBabel software was employed to convert these to MOL2 format. Molecular docking and analysis of the results of the interaction between the receptors and ligands were carried out using AutoDock and PyMOL software. Affinity ≤  − 5.0 kcal/mol indicated a good binding activity, and ≤  − 7.0 kcal/mol indicated a strong binding activity between the receptor and the ligand^39^. The lower the docking binding energy value, the higher the affinity between the component and the target protein.

### Viruses and cell lines

The PEDV used in this study was generously provided by Associate Professor Zhang Ding from Shanxi Agricultural University in Taigu, China. Vero and IPEC-J2 cell lines, obtained from BNCC in Beijing, China, were cultured, passaged, and stored according to established protocols. Vero cells were utilized for the propagation of PEDV, with the viral culture containing 10 μg/mL trypsin. A virus titer of 10^5.6^ TCID_50_/mL was determined using the Reed–Muench method. Subsequently, the multiplicity of infection (MOI) was computed using the formula PFU = 0.7 × TCID_50_.

### Cytotoxicity assay

IPEC-J2 cells were evenly distributed in a 96-well plate and cultured until 80–90% confluence. The cells were treated with various concentrations of MGQD. A preliminary test was conducted to establish the maximum allowable dose of MGQD, setting the upper limit at 20 mg/mL. MGQD was double diluted using DMEM (Gibco, USA), resulting in seven concentrations (20, 10, 5, 2.5, 1.25, 0.625, and 0.313 mg/mL).

After a 24-h incubation period, the cells were washed twice with PBS (Solarbio, China), and 100 μL of DMEM containing 10% CCK8 (Glpbio, USA) solution was added to each well. This was followed by hatch for one hour at 37 °C. The optical density at 450 nm was measured utilizing a microplate reader (Molecular Devices, USA). Cell viability ratios were determined following the guidelines provided by the CCK-8 manufacturer. In addition, GraphPad Prism™ 5.0 (GraphPad, USA) software was utilized to calculate the drug concentration associated with a 50% cell survival rate (TC50).

Guided by the results of the cytotoxicity assay, MGQD was diluted to seven concentrations (2.5, 1.25, 0.625, 0.313, 0.156, 0.078, and 0.039 mg/mL). The appropriate concentration of the drug for cells infected with PEDV (MOI = 0.1) was determined via microscopy and the CCK-8 method as described above.

Microscopy and RT‒qPCR detection of the anti-PEDV effect of MGQD.

Upon the cells reaching 80–90% confluence in a 12-well plate, the experiment was conducted using three distinct modes:

- (i) Viricidal Assay: MGQD (1.25 mg/mL) was mixed with PEDV (MOI = 0.2) in equal proportions to maintain the same final concentration of MGQD (0.625 mg/mL) and PEDV (MOI = 0.1) between viricidal and attachment, biosynthesis assays, followed by a two-hour incubation at 37 °C. Subsequently, a mixture containing 3 μg/mL trypsin was added (100 μL per well) and incubated for an additional two hours at 37 °C. The cells were then washed twice with PBS. Each well received an addition of 100 μL of DMEM containing 3 μg/mL trypsin.
- (ii) Attachment Assay: Prior to a two-hour infection with PEDV (MOI = 0.1), the cells were pretreated with MGQD (0.625 mg/mL) at 37 °C for four hours. After the fluid was discarded, the wells were washed twice, followed by the addition of DMEM containing 3 μg/mL trypsin.
- (iii) Biosynthesis Assay: Following a two-hour infection with PEDV (MOI = 0.1), the virus was removed, and the cells were washed twice. MGQD (0.625 mg/mL) containing 3 μg/mL trypsin was added to the cells for further incubation.

Two control groups were established for each mode: a control group (C) represented normal cells in control wells, and a model group (M) represented virus control wells. Each group comprised three replicates. The cells were collected after incubation at 37 °C for 24 h. Total RNA (1 μg) from IPEC-J2 cells was extracted utilizing RNA Isolater Total RNA Extraction Reagent (Vazyme, Nanjing, China) and converted into cDNA with HiScript II Q RT SuperMix for qPCR (+ gDNA wiper) (Vazyme). The resulting product was amplified using an AceQ Universal SYBR qPCR Master Mix (Vazyme) on a QuantStudio 6 Flex Real-Time PCR System (Thermo Fisher Scientific, USA). The mRNA levels were normalized to that of β-actin. A relative quantitative analysis of the data was conducted, enabling the calculation of the relative expression level of the PEDV gene via the 2^–ΔΔCt^ method. The primer sequences are listed in Table S9.

Detection of IFN-α, IFN-β, IFN-λ3, and TNF-α expression levels in IPEC-J2 cells.

In this bioassay, cells were incubated at 37 °C for 24 h. This was carried out in both six-well plates and 12-well plates. The relative numbers of mRNA copies of IFN-α, IFN-β, IFN-λ3, and TNF-α in IPEC-J2 cells were assessed through RT‒qPCR using the methodology described earlier.

Both the cell culture supernatant and the cellular components were collected to investigate any potential alterations in the levels of IFN-α, IFN-β, IFN-λ3, and TNF-α proteins. To achieve this, a Porcine IFN-α ELISA Kit (Solarbio, Cat#: SEKP-0045), Porcine IFN-β ELISA Kit (Solarbio, Cat#: SEKP-0046), Porcine IFN-λ3 ELISA Kit (MLBIO Biotechnology, Cat#: ml990071V, Shanghai, China), and Porcine TNF-α ELISA Kit (Solarbio, Cat#: SEKP-0009) were employed following the respective manufacturer’s instructions.

### Statistical analysis

The data are expressed as the mean ± SEM. Statistical analysis was performed via one-way analysis of variance (ANOVA) and Student’s t-test conducted with GraphPad Prism 5.0 software. Statistical significance was considered as a P value < 0.05.

### Ethics

This study involving animal subjects adhered to rigorous ethical standards and was conducted in strict accordance with the ARRIVE guidelines. All experimental procedures were approved by the Biological and Medical Ethics Committee of Xinxiang University (Approval Number: XYLL-22–024). The welfare and ethical treatment of animals were paramount throughout the research process. Measures were taken to minimize any potential discomfort, distress, or harm to the animals, and efforts were made to ensure the humane treatment of the animals during the entire study period. The research team remained vigilant in upholding ethical standards to contribute valuable insights while prioritizing animal welfare and regulatory compliance.

### Supplementary Information

Supplementary Table S1.

Supplementary Table S2.

Supplementary Table S3.

Supplementary Table S4.

Supplementary Table S5.

Supplementary Table S6.

Supplementary Table S7.

Supplementary Table S8.

Supplementary Table S9.

## Data Availability

The data presented in this study are available within the manuscript or supplementary information files.

## Abbreviations

CPEs: Cytopathological effects
FCM: Flow cytometry
GQD: Gegen Qinlian Decoction
H&E: Hematoxylin and eosin
IFN: Interferon
IPEC-J2: Intestinal porcine epithelial cell line-J2
MGQD: Modified Gegen Qinlian decoction
MLNs: Mesenteric lymph nodes
MOI: Multiplicity of infection
PED: Porcine epidemic diarrhea
PEDV: Porcine epidemic diarrhea virus
PPs: Peyer's patches
TNF: Tumour necrosis factor
