# Supplementary material for: Integrating network pharmacology with pharmacological research to elucidate the mechanism of modified Gegen Qinlian Decoction in treating porcine epidemic diarrhea
Source: Sci Rep. 2024 Aug 15;14:18929. doi: 10.1038/s41598-024-70059-5 (PMC11327325; doi:10.1038/s41598-024-70059-5)
Supplement: Supplementary file 1 — Supplementary Table S1. [file 41598_2024_70059_MOESM1_ESM.docx]

**Table S1.** Features of identified components of MGQD by UHPLC-MS/MS.

| No. | Compound | PubChem CID | Class | herbSet | mzmed | rtmed | ppm | ms2Adduct | Composite  Score |
| --- | --- | --- | --- | --- | --- | --- | --- | --- | --- |
| 1 | 6,7-Dihydroxycoumarin | [5281416](https://pubchem.ncbi.nlm.nih.gov/compound/5281416) | Phenylpropanoids | AY | 179.0339 | 148.5855 | 0.4334 | [M+H]+ | 0.9785 |
| 2 | Ferulic acid | [445858](https://pubchem.ncbi.nlm.nih.gov/compound/445858) | Phenylpropanoids | HL | 195.0651 | 204.9385 | 0.3788 | [M+H]+ | 0.9772 |
|  |  |  |  |  | 193.0505 | 322.8475 | 2.3494 | [M-H]- | 0.9654 |
| 3 | Umbelliferone | [5281426](https://pubchem.ncbi.nlm.nih.gov/compound/5281426) | Phenylpropanoids | AY | 163.0391 | 302.549 | 0.8078 | [M+H]+ | 0.9961 |
|  |  |  |  |  | 161.0246 | 302.4525 | 2.5849 | [M-H]- | 0.9191 |
| 4 | Liquiritin | [503737](https://pubchem.ncbi.nlm.nih.gov/compound/503737) | Flavonoids | GC | 419.1334 | 337.1685 | 1.4353 | [M+H]+ | 0.8511 |
| 5 | Quercetin | [5280343](http://pubchem.ncbi.nlm.nih.gov/summary/summary.cgi?cid=5280343) | Flavonoids | HL,GC, QI, AY | 303.0496 | 338.4965 | 1.4412 | [M+H]+ | 1 |
|  |  |  |  |  | 301.0353 | 448.548 | 1.0063 | [M-H]- | 0.9483 |
| 6 | Oxyberberine | [11066](https://pubchem.ncbi.nlm.nih.gov/compound/11066) | Alkaloid | HL | 352.1182 | 342.607 | 0.4657 | [M+H]+ | 0.9084 |
| 7 | Puerarin | [5281807](http://pubchem.ncbi.nlm.nih.gov/summary/summary.cgi?cid=5281807) | Flavonoids | GG | 417.1185 | 386.981 | 1.3111 | [M+H]+ | 0.8494 |
| 8 | Kaempferol | [5280863](http://pubchem.ncbi.nlm.nih.gov/summary/summary.cgi?cid=5280863) | Flavonoids | GC,QI | 287.0550 | 388.664 | 0.1650 | [M+H]+ | 0.9999 |
|  |  |  |  |  | 285.0406 | 540.4925 | 1.2378 | [M-H]- | 0.9969 |
| 9 | Jatrorrhizine | [72323](https://pubchem.ncbi.nlm.nih.gov/compound/72323) | Alkaloid | HL | 338.1388 | 397.046 | 0.5307 | [M+] | 0.9065 |
| 10 | Berberine | [2353](https://pubchem.ncbi.nlm.nih.gov/compound/2353) | Alkaloid | HL | 336.1235 | 397.6695 | 1.4393 | [M+H]+ | 0.7984 |
| 11 | Columbamine | [72310](https://pubchem.ncbi.nlm.nih.gov/compound/72310) | Alkaloid | HL | 339.1415 | 399.853 | 1.4240 | [M+H]+ | 0.9042 |
| 12 | Liquiritigenin | [114829](http://pubchem.ncbi.nlm.nih.gov/summary/summary.cgi?cid=114829) | Flavonoids | GC | 257.0806 | 413.642 | 1.3804 | [M+H]+ | 0.9970 |
|  |  |  |  |  | 255.0667 | 543.639 | 2.8589 | [M-H]- | 0.9908 |
| 13 | Isorhamnetin | [5281654](http://pubchem.ncbi.nlm.nih.gov/summary/summary.cgi?cid=5281654) | Flavonoids | GC,QI | 317.0661 | 416.8095 | 0.3920 | [M+H]+ | 0.9999 |
|  |  |  |  |  | 315.0512 | 518.314 | 0.6620 | [M-H]- | 0.8014 |
| 14 | Chrysin | [5281607](https://pubchem.ncbi.nlm.nih.gov/compound/5281607) | Flavonoids | HQ | 255.0649 | 467.491 | 0.5288 | [M+H]+ | 1 |
|  |  |  |  |  | 253.0506 | 467.14 | 1.6755 | [M-H]- | 0.9995 |
| 15 | Calycosin | [5280448](https://pubchem.ncbi.nlm.nih.gov/compound/5280448) | Flavonoids | QI | 285.0759 | 485.8225 | 0.3948 | [M+H]+ | 1 |
| 16 | Coumestrol | [5281707](https://pubchem.ncbi.nlm.nih.gov/compound/5281707) | Phenylpropanoids | GG | 269.0448 | 487.163 | 0.8819 | [M+H]+ | 0.9275 |
| 17 | Wogonoside | [3084961](https://pubchem.ncbi.nlm.nih.gov/compound/3084961) | Flavonoids | HQ | 461.1081 | 538.776 | 0.2736 | [M+H]+ | 0.9060 |
|  |  |  |  |  | 459.0927 | 520.108 | 0.5491 | [M-H]- | 0.8572 |
| 18 | Formononetin | [5280378](http://pubchem.ncbi.nlm.nih.gov/summary/summary.cgi?cid=5280378) | Flavonoids | GG,GC, QI | 269.0806 | 559.6475 | 1.3573 | [M+H]+ | 0.9371 |
|  |  |  |  |  | 267.0663 | 238.6515 | 0.9738 | [M-H]- | 0.9907 |
| 19 | Glycyrol | [5320083](https://pubchem.ncbi.nlm.nih.gov/compound/5320083) | Phenylpropanoids | GC | 367.1176 | 563.559 | 1.1099 | [M+H]+ | 0.8330 |
| 20 | Baicalin | [64982](https://pubchem.ncbi.nlm.nih.gov/compound/64982) | Flavonoids | HQ | 447.0915 | 581.905 | 1.1604 | [M+H]+ | 0.9154 |
| 21 | 18 beta-Glycyrrhetintic Acid | [10114](https://pubchem.ncbi.nlm.nih.gov/compound/10114) | Terpenoids | GC | 471.3477 | 583.888 | 0.6287 | [M+H]+ | 0.8462 |
| 22 | Daidzein | 5281708 | Flavonoids | GG | 255.0654 | 608.6445 | 1.6991 | [M+H]+ | 1 |
|  |  |  |  |  | 253.0509 | 429.744 | 0.2738 | [M-H]- | 1 |
| 23 | [Gancaonin A](https://old.tcmsp-e.com/molecule.php?qn=4856) | [5317478](https://pubchem.ncbi.nlm.nih.gov/compound/5317478) | Flavonoids | GC | 353.1376 | 609.0115 | 1.0716 | [M+H]+ | 0.8230 |
|  |  |  |  |  | 351.1244 | 705.6445 | 1.1625 | [M-H]- | 0.8174 |
| 24 | Isolicoflavonol | [5318585](https://pubchem.ncbi.nlm.nih.gov/compound/5318585) | Flavonoids | GC | 355.1171 | 650.22 | 2.5513 | [M+H]+ | 0.7618 |
| 25 | Palmatine | [19009](https://pubchem.ncbi.nlm.nih.gov/compound/19009) | Alkaloid | HL | 352.1539 | 659.64 | 0.2935 | [M+] | 0.9981 |
| 26 | Licochalcone A | [5318998](http://pubchem.ncbi.nlm.nih.gov/summary/summary.cgi?cid=5318998) | Flavonoids | GC | 339.1596 | 667.205 | 1.7342 | [M+H]+ | 0.9411 |
| 27 | Baicalein | [5281605](http://pubchem.ncbi.nlm.nih.gov/summary/summary.cgi?cid=5281605) | Flavonoids | HQ | 271.0601 | 681.851 | 0.5520 | [M+H]+ | 0.9861 |
|  |  |  |  |  | 269.0459 | 622.2055 | 0.1951 | [M-H]- | 1 |
| 28 | Genistein | [5280961](https://pubchem.ncbi.nlm.nih.gov/compound/5280961) | Flavonoids | GG | 271.0596 | 734.227 | 1.3789 | [M+H]+ | 1 |
|  |  |  |  |  | 269.0459 | 385.325 | 0.5022 | [M-H]- | 0.9928 |
| 29 | Isofraxidin | [5318565](https://pubchem.ncbi.nlm.nih.gov/compound/5318565) | Phenylpropanoids | AY | 223.0603 | 774.7205 | 1.2673 | [M+H]+ | 0.9909 |
|  |  |  |  |  | 221.0459 | 226.62 | 0.2391 | [M-H]- | 0.7633 |
| 30 | Skullcapflavone II | [124211](http://pubchem.ncbi.nlm.nih.gov/summary/summary.cgi?cid=124211) | Flavonoids | HQ | 375.1077 | 782.451 | 1.8031 | [M+H]+ | 0.8602 |
| 31 | Wogonin | [5281703](http://pubchem.ncbi.nlm.nih.gov/summary/summary.cgi?cid=5281703) | Flavonoids | HQ | 285.0762 | 1657.65 | 0.6378 | [M+H]+ | 1 |
|  |  |  |  |  | 283.0612 | 603.7215 | 0.7393 | [M-H]- | 1 |
| 32 | Scopoletin | [5280460](https://pubchem.ncbi.nlm.nih.gov/compound/5280460) | Phenylpropanoids | AY | 193.0494 | 1665.8 | 2.0677 | [M+H]+ | 1 |
| 33 | Betaine | [247](https://pubchem.ncbi.nlm.nih.gov/compound/247) | Alkaloid | QI | 118.0861 | 1792.48 | 0.9474 | [M+H]+ | 0.9858 |
| 34 | Sucrose | [5988](https://pubchem.ncbi.nlm.nih.gov/compound/5988) | Organooxygen compounds | QI | 341.1081 | 80.1466 | 2.5725 | [M-H]- | 0.9204 |
| 35 | Daidzin | [107971](https://pubchem.ncbi.nlm.nih.gov/compound/107971) | Flavonoids | GG | 451.0882 | 309.547 | 18.1095 | [M+Cl]- | 0.7962 |
| 36 | 2-(2,6-Dihydroxyphenyl)-3,5,7- trihydroxychromen-4-one | [5320471](https://pubchem.ncbi.nlm.nih.gov/compound/5320471) | Flavonoids | HQ | 301.0353 | 318.007 | 1.0592 | [M-H]- | 0.8813 |
| 37 | Oroxylin A | [5320315](http://pubchem.ncbi.nlm.nih.gov/summary/summary.cgi?cid=5320315) | Flavonoids | HQ | 283.0611 | 343.467 | 0.5143 | [M-H]- | 0.9815 |
| 38 | Scutellarin | [185617](https://pubchem.ncbi.nlm.nih.gov/compound/185617) | Flavonoids | HQ | 461.0737 | 348.593 | 1.4153 | [M-H]- | 0.8232 |
| 39 | Genistin | [5281377](https://pubchem.ncbi.nlm.nih.gov/compound/5281377) | Flavonoids | GG | 431.0976 | 385.181 | 0.9994 | [M-H]- | 0.8844 |
| 40 | Isoliquiritin | [5318591](https://pubchem.ncbi.nlm.nih.gov/compound/5318591) | Chalcones | GC | 417.1185 | 418.3115 | 1.1894 | [M-H]- | 0.9351 |
| 41 | Naringenin | [932](https://pubchem.ncbi.nlm.nih.gov/compound/932) | Flavonoids | AY | 271.0616 | 495.215 | 2.0521 | [M-H]- | 0.9412 |
| 42 | Hispidulin | [5281628](https://pubchem.ncbi.nlm.nih.gov/compound/5281628) | Flavonoids | AY | 299.0557 | 502.916 | 1.1474 | [M-H]- | 0.9786 |
| 43 | Glabridin | [124052](http://pubchem.ncbi.nlm.nih.gov/summary/summary.cgi?cid=124052) | Flavonoids | GC | 323.1298 | 699.5835 | 2.3590 | [M-H]- | 0.8424 |
