# Supplementary material for: Integrating network pharmacology with pharmacological research to elucidate the mechanism of modified Gegen Qinlian Decoction in treating porcine epidemic diarrhea
Source: Sci Rep. 2024 Aug 15;14:18929. doi: 10.1038/s41598-024-70059-5 (PMC11327325; doi:10.1038/s41598-024-70059-5)
Supplement: Supplementary file 7 — Supplementary Table S7. [file 41598_2024_70059_MOESM7_ESM.docx]

**Table S7.** The affinity of core components of MGQD and key targets (Kcal/mol).

|  | IL6 (6MG1) | EGFR (8A27) | CXCL10 (8K2X) | STAT1 (3SU6) | IFNB1 (8BTJ) | CXCL8 (6LFL) | FOS (6S32) | TNF (1VYR) |
| --- | --- | --- | --- | --- | --- | --- | --- | --- |
| [CID5280961](https://pubchem.ncbi.nlm.nih.gov/compound/5280961) | -7.4 | -7 | -6.9 | -6.5 | -8.8 | -6.8 | -7.5 | -6.3 |
| CID5281708 | -7.1 | -6.6 | -6.1 | -7.1 | -8.4 | -5.8 | -6.7 | -6.2 |
| CID5281807 | -7.9 | -7.2 | -6.9 | -6.2 | -7 | -6.2 | -7.5 | -6.8 |
| [CID5281607](https://pubchem.ncbi.nlm.nih.gov/compound/5281607) | -8.2 | -7.4 | -7.2 | -7.2 | -8.7 | -7.9 | -7.5 | -8.2 |
| CID5281703 | -8.3 | -7 | -6.2 | -7.1 | -7.7 | -6.5 | -7.5 | -8.2 |
| CID5281605 | -8.1 | -7.5 | -6.6 | -6.6 | -9 | -6 | -8.5 | -8.9 |
| CID19009 | -7 | -6.6 | -6.5 | -6.5 | -7.6 | -5.2 | -6.7 | -7.6 |
| CID72323 | -6.6 | -6.6 | -6.7 | -6.7 | -8.2 | -5.4 | -5.9 | -6.9 |
| CID2353 | -8 | -8.7 | -7.4 | -7 | -8.5 | -7 | -7.5 | -8.5 |
| CID114829 | -8.2 | -7.4 | -6.6 | -6.3 | -6.4 | -5.8 | -6.8 | -8.5 |
| CID5318998 | -6.4 | -6.7 | -5.9 | -5.9 | -5.7 | -6.1 | -6.5 | -8.2 |
| CID5320083 | -8.7 | -9.7 | -9.4 | -7.1 | -7.8 | -7.4 | -8.9 | -10.6 |
| CID124052 | -7.9 | -8.1 | -8 | -6.8 | -7.5 | -6.6 | -7.3 | -8.4 |
| CID5280448 | -8.2 | -7.9 | -6.8 | -6.9 | -7.6 | -7.4 | -7.4 | -8.5 |
| CID5281416 | -7.9 | -7.8 | -7 | -6.6 | -8 | -6.1 | -7 | -8.4 |
| CID5281628 | -8.1 | -9 | -7.4 | -6.8 | -8.5 | -7.1 | -9 | -8.7 |
| CID932 | -8.6 | -10.6 | -8.9 | -7.7 | -7.9 | -6.9 | -8.7 | -9.9 |
| CID5280378 | -7.8 | -6.6 | -6.8 | -5.7 | -8.8 | -6.6 | -6.2 | -8.4 |
| [CID5280343](http://pubchem.ncbi.nlm.nih.gov/summary/summary.cgi?cid=5280343) | -7.6 | -6.7 | -6.2 | -6.6 | -8.2 | -6.4 | -7.9 | -8.2 |
| CID5280863 | -5.9 | -6.4 | -5.9 | -5.6 | -6.6 | -5.1 | -5.7 | -6.9 |
| CID5281654 | -7.4 | -6.1 | -5.7 | -5.8 | -5.8 | -5.7 | -6.1 | -8.7 |
