# Supplementary material for: Integrating network pharmacology with pharmacological research to elucidate the mechanism of modified Gegen Qinlian Decoction in treating porcine epidemic diarrhea
Source: Sci Rep. 2024 Aug 15;14:18929. doi: 10.1038/s41598-024-70059-5 (PMC11327325; doi:10.1038/s41598-024-70059-5)
Supplement: Supplementary file 8 — Supplementary Table S8. [file 41598_2024_70059_MOESM8_ESM.docx]

**Table S8.** Medicinal plants composition of modified Gegen Qinlian decoction and Gegen Qinlian decoction.

| Pharmaceutical  name | Family | Latin name | Chinese  name | Abbr | Weight  ratio | MGQD | GQD |
| --- | --- | --- | --- | --- | --- | --- | --- |
| Puerariae Lobatae Radix | [Fabaceae](https://worldfloraonline.org/taxon/wfo-7000000323) | *Pueraria montana var. lobata* (Willd.) Maesen & S.M.Almeida ex Sanjappa & Predeep | Gegen | GG | 5 | + | + |
| Scutellariae Radix | Lamiaceae | [*Scutellaria* *baicalensis* Georgi](http://www.theplantlist.org/tpl1.1/record/kew-188938) | Huangqin | HQ | 3 | + | + |
| Coptidis Rhizoma | Ranunculaceae | [*Coptis* *chinensis* Franch.](http://www.theplantlist.org/tpl1.1/record/kew-2736105) | Huanglian | HL | 3 | + | + |
| Glycyrrhizae Radix Et Rhizoma | [Fabaceae](https://worldfloraonline.org/taxon/wfo-7000000323) | Glycyrrhiza uralensis Fisch. ex DC. | Gancao | GC | 2 | + | + |
| Astragali Radix | [Fabaceae](https://worldfloraonline.org/taxon/wfo-7000000323) | [*Astragalus membranaceus* Fisch.ex Bunge](http://www.theplantlist.org/tpl1.1/record/ild-32156) | Huangqi | QI | 3 | + | - |
| Artemisiae Argyi Folium | [Asteraceae](https://worldfloraonline.org/taxon/wfo-7000000146) | [*Artemisia argyi*H.Lév. & Vaniot](http://www.theplantlist.org/tpl1.1/record/gcc-151027) | Aiye | AY | 3 | + | - |

“+” represents with herb added. “-” represents without herb added.
