# Supplementary material for: Integrating network pharmacology with pharmacological research to elucidate the mechanism of modified Gegen Qinlian Decoction in treating porcine epidemic diarrhea
Source: Sci Rep. 2024 Aug 15;14:18929. doi: 10.1038/s41598-024-70059-5 (PMC11327325; doi:10.1038/s41598-024-70059-5)
Supplement: Supplementary file 9 — Supplementary Table S9. [file 41598_2024_70059_MOESM9_ESM.docx]

**Table S9.** Primer sequences for qRT-PCR.

| Gene name | Forward primer | Reverse primer |
| --- | --- | --- |
| β-actin | GGCTCAGAGCAAGAGAGGTATCC | GGTCTCAAACATGATCTGAGTCATCT |
| PEDV | GCTTGCTTCGGACCCAGAGG | ACGAACAGCCACATTACCACCA |
| TNF-α | TGGCCCCTTGAGCATCA | CGGGCTTATCTGAGGTTTGAGA |
| IFN-α | GCCTCCTGCACCAGTTCTACA | TGCATGACACAGGCTTCCA |
| IFN-β | GCTAACAAGTGCATCCTCCAAA | CCAGGAGCTTCTGACATGCCA |
| IFN-λ3 | ACATCCACGTCGAACTTCAGGCT | GATGTGCAAGTCTCCACTGGT |
